# Discovery of pancreastatin inhibitor PSTi8 for the treatment of insulin resistance and diabetes: studies in rodent models of diabetes mellitus

**DOI:** 10.1038/s41598-018-27018-8

**Published:** 2018-06-07

**Authors:** Zakir Hossain, Guru R. Valicherla, Anand P. Gupta, Anees A. Syed, Mohammed Riyazuddin, Sharat Chandra, Mohammad I. Siddiqi, Jiaur R. Gayen

**Affiliations:** 1 Pharmaceutics & Pharmacokinetics Division, CSIR-Central Drug Research Institute, Lucknow, 226031 India; 2 Molecular and Structural Biology Division, CSIR-Central Drug Research Institute, Lucknow, 226031 India; 3 Academy of Scientific and Innovative Research (AcSIR), New Delhi, India

## Abstract

Pancreastatin (PST) is an endogenous peptide which regulates glucose and lipid metabolism in liver and adipose tissues. In type 2 diabetic patients, PST level is high and plays a crucial role in the negative regulation of insulin sensitivity. Novel therapeutic agents are needed to treat the diabetes and insulin resistance (IR) against the PST action. In this regard, we have investigated the PST inhibitor peptide-8 (PSTi8) action against diabetogenic PST. PSTi8 rescued PST-induced IR in HepG2 and 3T3L1 cells. PSTi8 increases the GLUT4 translocation to cell surface to promote glucose uptake in L6-GLUT4*myc* cells. PSTi8 treatment showed an increase in insulin sensitivity in db/db, high fat and fructose fed streptozotocin (STZ) induced IR mice. PSTi8 improved the glucose homeostasis which is comparable to metformin in diabetic mice, characterized by elevated glucose clearance, enhanced glycogenesis, enhanced glycolysis and reduced gluconeogenesis. PST and PSTi8 both were docked to the GRP78 inhibitor binding site in protein-protein docking, GRP78 expression and its ATPase activity studies. The mechanism of action of PSTi8 may be mediated by activating IRS1/2-phosphatidylinositol-3-kinase-AKT (FoxO1, Srebp-1c) signaling pathway. The discovery of PSTi8 provides a promising therapeutic agent for the treatment of metabolic diseases mainly diabetes.

## Introduction

The Chromogranin A (Chga) is a pro-protein present in secretory cells of neuroendocrine system. Upon proteolysis, Chga gives rise to various bioactive peptides like pancreastatin (PST), vasostatin, catestatin, parastatin and chromacin^1^. PST (CHGA_250–301_)^2–4^ is an endogenous peptide which regulates the glucose, protein and lipid metabolism by inhibiting glucose uptake, protein synthesis and increasing spillover of fatty acid in adipose and liver tissue^5,6^. PST metabolic effects have been confirmed in humans and it level is elevated in diabetic population^3,7,8^. Moreover PST has three naturally occurring variants with rank order of efficacy to inhibit insulin-stimulated glucose-uptake and diabetes progression:PST-G297S > PST-E287K > PST-Wt^9^. PST treatment also inhibits glycogen synthesis and stimulates gluconeogenesis by decreasing phosphorylation of insulin receptor substrate (IRS) at tyrosine residue, hence showing anti-insulin effect in hepatocytes^10^. These profound effects are mediated by activation of receptor signaling system that belongs to the several spanning transmembrane receptor coupled to Gq-PLCβ-calcium-PKC signaling pathway^11,12^. Bandyopadhyay *et al*. reported PSTv1 (pancreastatin variant) action on improved glucose tolerance in WT-DIO mice & *Chga* KO mice with normal chow diet^13^.

Due to anti-insulin effect of PST, it stimulates diabetic complication and disrupt glucose and lipid metabolism. We hypothesized that PSTi8 may inhibit PST effect and improve glucose and lipid homeostasis by modulating PLC AKT-FoxO1 and Srebp-1c pathway.

## Results

### Pancreastatin inhibitor rescued IR *in vitro*

**Figure 1:**
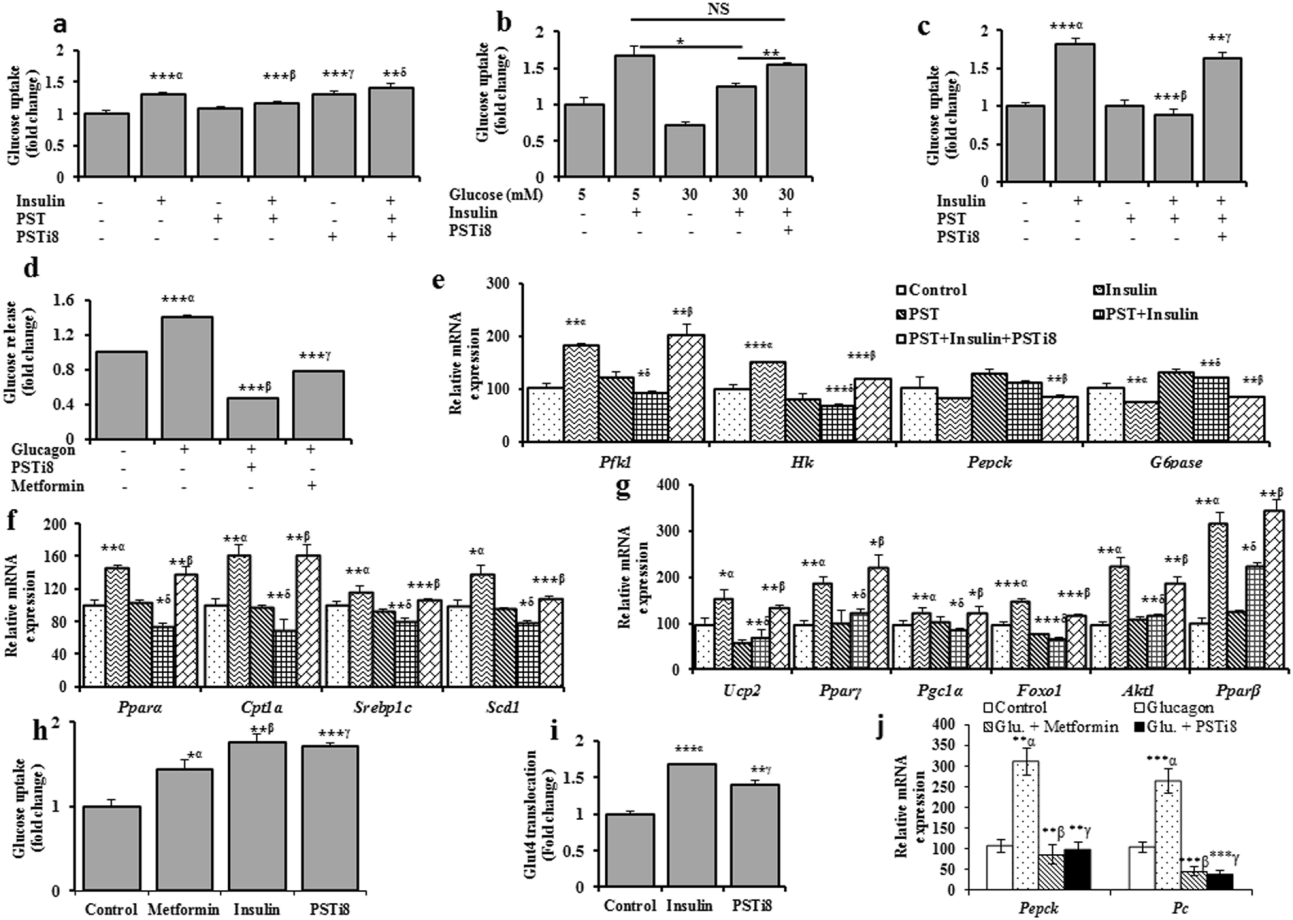
Pancreastatin inhibitor rescued IR *in vitro*. (**a**) PSTi8 attenuated PST induced IR in HepG2 cells. α, control vs insulin (100 nM); β, insulin vs PST (25 nM) + insulin; γ, control vs PSTi8 (150 nM) and δ, insulin +PST vs insulin + PST + PSTi8. (**b**) PSTi8 controlled the high-glucose induced IR in HepG2 cells. (**c**) PSTi8 rescued PST elevated IR in 3T3L1 cells. α, control vs insulin (100 nM); β, insulin vs PST (200 nM) + insulin and γ, insulin + PST vs insulin + PST + PSTi8 (800 nM). (**d**) PSTi8 inhibits the glucose release in the presence of glucagon in HepG2 cells. α, control vs glucagon (6 nM); β, glucagon vs glucagon +PSTi8 (150 nM); γ, glucagon vs glucagon + metformin (100 µM). (**e**) Effect of insulin, PST and PSTi8 treatment on expression of glycolysis genes (*Hk*, *Pfk1*) and gluconeogenesis genes (*Pepck* and *G6pase*) in HepG2 cells. α, control vs insulin (100 nM); δ, insulin vs PST (25 nM) + insulin; β, PST + insulin vs PST + insulin + PSTi8 (150 nM). (**f**) Effect of insulin, PST and PSTi8 treatment on expression of lipogenic genes (*Pparα*, *Cpt1a*, *Srebp-1c* and *Scd1*) in HepG2 cells. α, control vs insulin (100 nM); β, PST (25 nM) + insulin vs PST + insulin + PSTi8 (150 nM). (**g**) Effect of insulin, PST and PSTi8 treatment on expression of lipogenic genes (*Ucp2*, *Pparγ*, *Pgc1α*, *FoxO1*, *Akt1* and *Pparβ*) in 3T3L1 cells. α, control vs insulin (100 nM); δ, insulin vs PST (25 nM) + insulin; β, insulin vs PST (25 nM) + insulin; γ, PST + insulin vs PST + insulin + PSTi8 (150 nM). All genes are normalized to *Gapdh* and *β*-*actin* as reference genes in HepG2 and 3T3L1 cells, respectively. (**h**) Effect of PSTi8 on glucose uptake in L6 cells. α, control vs metformin (10 µM); β, control vs insulin (100 nM); γ, control vs PSTi8 (100 nM). (**i**) Effect of PSTi8 (150 nM) and insulin (200 nM) on GLUT4 translocation to surface in L6-GLUT4*myc* cells. (**j**) PSTi8 inhibits gluconeogenic gene (*Pepck*, *Pc*) expression in glucagon stimulated HepG2 cells. α, control vs glucagon (6 nM); β glucagon vs glu. + metformin (100 µM) and γ glucagon vs glu. + PSTi8 (150 nM). *P < 0.05; **P < 0.01; ***P < 0.001, NS, Non-significant. Error bar indicate mean ± s.e.m.

To determine does PSTi8 inhibit PST or high glucose induce IR and stimulate insulin effect on glucose uptake. In HepG2 and 3T3L1 cells^14^, PST was found to inhibit the insulin action. PST inhibitory effect of PSTi8 was observed in the presence of PST and insulin (Fig. 1a and c). Individual effect of PSTi8 on glucose uptake was also found in the absences of PST and insulin. (Fig. 1a) Instead of PST induce IR, PSTi8 also stimulate glucose uptake in IR-HepG2 cells which was developed by providing high D-glucose for 48 h^15^ (Fig. 1b). PSTi8 showed antidiabetic activity like metformin by significantly enhancing glucose uptake in L6 cells in comparison to control (Fig. 1h). The increase in glucose uptake in L6 cell on PSTi8 treatment occurs through the increase in translocation of GLUT4 on cell surface (Fig. 1i). The increased effect on glucose uptake of PSTi8 also regulate gluconeogenesis, this was examined in HepG2 cells. PSTi8 effectively antagonizes the enhanced effect of Glucagon on glucose production from lactate and pyruvate, which is comparable to metformin^16^ (Fig. 1d). To further evaluate PSTi8 effect on glycolysis, gluconeogenesis and lipogensis mRNA expressions were studied. PST inhibit the insulin effect and suppress peroxisome proliferator activator receptor (*Ppar*)*γ*, *uncoupling protein* 2 (*Ucp2*), *FoxO1*, pparγ coactivator-α (*Pgc1α*), *Akt1 and Pparβ* gene expression responsible for lipogensis and adipogensis which is significantly reversed by PSTi8 treatment in 3T3L1 cells^17,18^ (Fig. 1g). The stimulated effect on glucose uptake and suppression in gluconeogenesis was associated with increase in *Pfk and hexokinase* (*Hk*) and decrease in glucose-6-phosphatase (*G6pase*), *Pepck* and pyruvate carboxylase (*Pc*) gene expression on PSTi8 treatment in HepG2 cells (Fig. 1e,j). PSTi8 also modulate gene expression associated with lipogensis and fatty acid oxidation in HepG2 cells. On PSTi8 treatment, *Srebp-1c*, stearoyl-CoA desaturase-1 (*Scd1*) gene for lipogensis and *Pparα* and carnitine palmitoyl transferase-1a (*Cpt1a*) gene for uptake and oxidation of fatty acid get stimulated (Fig. 1f).

### PSTi8 attenuates IR in High Fat Diet (HFD)-fed, High Fructose Diet (HFrD)-fed and db/db diabetic mice and its effect is compared to metformin

**Figure 2:**
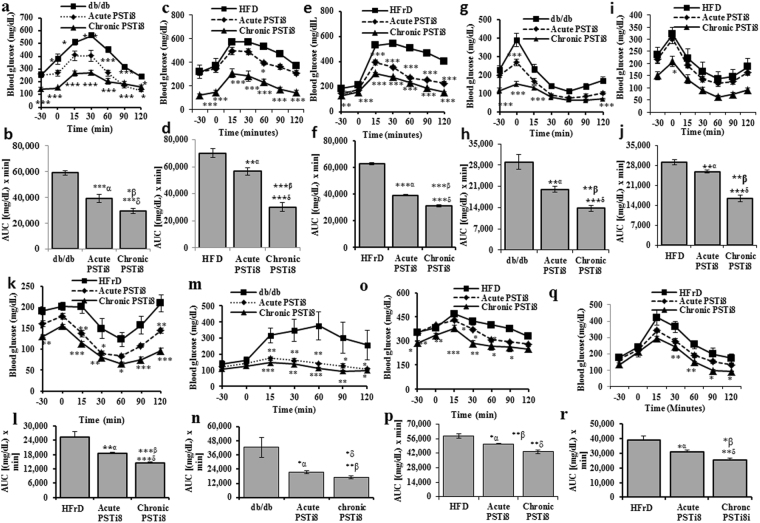
PSTi8 attenuates IR in HFD-fed, HFrD-fed and db/db diabetic mice. Acute (i.p, 5 mg/kg) and chronic (i.p, 2 mg/kg for 7 days), administration of PSTi8 during i.p GTT (0.5 g/kg) for db/db, (1 gram/kg) for HFD, HFrD mice or ITT (1 IU/kg) for db/db, (0.6 IU/kg) for HFD, HFrD mice or PTT (2 gram/kg) for db/db and HFD, HFrD in fasting (6 h) mice (n = 6). (**a**,**c**,**e**) i.p GTT; (**b**,**d**,**f**) AUC of i.p GTT; (**g**,**i**,**k**) i.p GTT; (**h**,**j**,**l**) AUC of i.p ITT; (**m**,**o**,**q**) i.p PTT; (**n**,**p**,**r**) AUC of i.p PTT. α, db/db, HFD, HFrD vs acute PSTi8 treatment, δ, db/db, HFD, HFrD vs chronic PSTi8 treatment and β, acute PSTi8 treated vs chronic PSTi8 treated db/db, HFD, HFrD. *P < 0.05; **P < 0.01; ***P < 0.001. Error bar indicate mean ± s.e.m.

To examine whether PSTi8 enhance glucose clearance, insulin sensitivity and attenuates gluconeogenesis in insulin resistance mice model, phenotyping screening was performed. PSTi8 treatment in HFD, HFrD and db/db mice significantly attenuated IR as compared to vehicle control. In ipGTT, PSTi8 acute (5 mg/kg) and chronic (2 mg/kg) treatment significantly control glucose homeostasis. Chronic PSTi8 showed more prominent improvement in glucose tolerance compared to acute PSTi8 in HFD fed (Fig. 2c,d), HFrD fed (Fig. 2e,f) and db/db (Fig. 2a,b) mice. Similarly same results were also seen in ipITT and ipPTT. In ipITT chronic and acute PSTi8 enhances insulin sensitivity compared to HFD fed (Fig. 2i,j), HFrD fed (Fig. 2k,l) and db/db (Fig. 2g,h) mice. But the effect of chronic PSTi8 is higher than the acute in affiliation to insulin sensitivity. In ipPTT chronic and acute PSTi8 treatment suppress gluconeogenesis (i.e hepatic glucose output) more compared to HFD fed (Fig. 2o,p), HFrD fed (Fig. 2q,r) and db/db (Fig. 2m,n) mice. The data values of GTT, ITT and PTT is mentioned in Table 1. The fasting blood glucose level of HFD, HFrd and db/db mice in chronic PSTi8 treatment (120.83 ± 13.02 mg/dL), (129 ± 4.06 mg/dL), (127.66 ± 10.37 mg/dL) were found significantly decreased in compared to untreated mice (316.33 ± 56.98 mg/dL), (185.33 ± 9.52 mg/dL) and (254.83 ± 20.63 mg/dL) (0 minute data of Fig. 2a,c,e) respectively.

**Table 1:** Anti-diabetic activity of PSTi8 on the basis of AUC in different animal models during GTT, ITT and PTT after acute and chronic treatment.

Phenotyping screening	Glucose tolerance test			Insulin tolerance test			Pyruvate tolerance test		
Treatment	saline	Acute PSTi8	Chronic PSTi8	saline	Acute PSTi8	Chronic PSTi8	Saline	Acute PSTi8	Chronic PSTi8
Mice model									
HFD (N = 6)	69980 ± 3317 mg/dL × min	56611 ± 2847 mg/dL × min, **α	30083 ± 3353 mg/dL × min, ***β, ***δ	29109 ± 820 mg/dL × min	25839 ± 546 mg/dL × min, **α	(16410 ± 1215 mg/dL × min, **β, ***δ	58582 ± 2019 mg/dL × min	51131 ± 612 mg/dL × min, *α	43481 ± 1856 mg/dL × min, **β, **δ
HFrD (N = 6)	62870 ± 822 mg/dL × min	38997 ± 581 mg/dL × min,***α	31023 ± 875 mg/dL × min, ***β, ***δ	23605 ± 1466 mg/dL × min	(18413 ± 480 mg/dL × min, **α	14608 ± 392 mg/dL × min, ***β, ***δ	38767 ± 3020 mg/dL × min	31142 ± 5926 mg/dL × min, *α	25323.8 ± 1492.98 mg/dL × min, *β, **δ
db/db (N = 6)	59271 ± 1750 mg/dL × min	38967 ± 3204 mg/dL × min, ***α	29356 ± 2101 mg/dL × min, *β, ***δ	29307 ± 2394 mg/dL × min	19983 ± 1004 mg/dL × min, *α	13832 ± 993 mg/dL × min, **β, ***δ	42401 ± 8387 mg/dL × min	21360 ± 1008 mg/dL × min, *α	17025 ± 1119 mg/dL × min, **β, *δ

(N = 6, mean ± s.e.m), α, saline vs acute PSTi8; β, saline vs chronic PSTi8 and δ, acute PSTi8 vs chronic PSTi8 *P < 0.05; **P < 0.01; ***P < 0.001.

**Figure 3:**
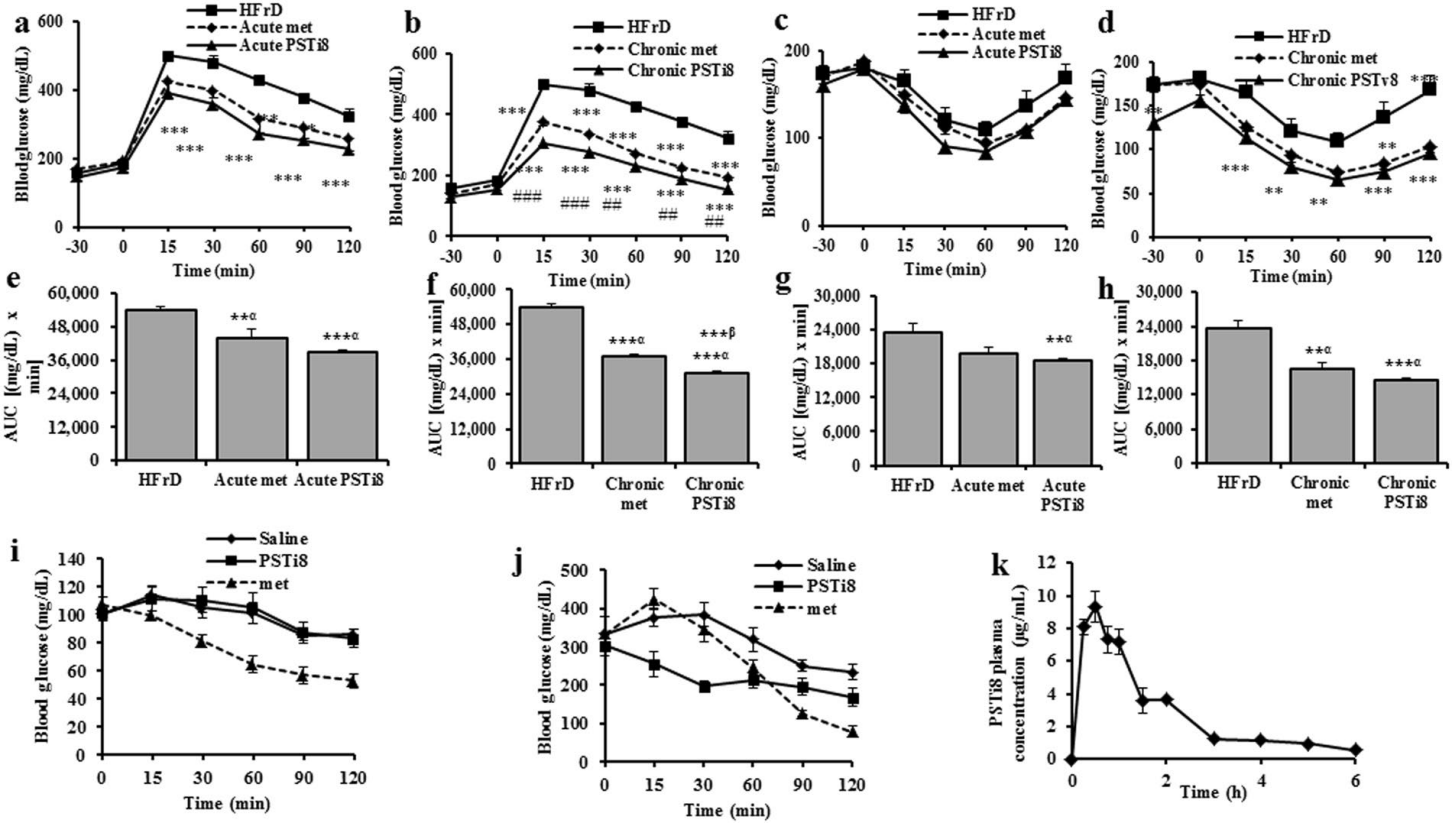
Efficacy of PSTi8 as compared to metformin. Acute PSTi8 (i.p, 5 mg/kg), chronic PSTi8 (i.p, 2 mg/kg for 7 days), acute metformin (p.o, 300 mg/kg) and chronic metformin (p.o, 300 mg/kg for 7 days) administration (30 min before bolus glucose/insulin) during i.p GTT (1 g/kg) or ITT (0.6 IU/kg) in fasting (6 h) HFrD mice (n = 6). (**a**) i.pGTT with acute metformin and PSTi8; (**b**) i.pGTT with chronic metformin and PSTi8; (**c**) i.p ITT with acute metformin and PSTi8; (**d**) i.p ITT with chronic metformin and PSTi8; (**e**) AUC of i.p GTT with acute metformin and PSTi8; (**f**) AUC of i.p GTT with chronic metformin and PSTi8; (**g**) AUC of i.p ITT with acute metformin and PSTi8; (**h**) AUC of i.p ITT with chronic metformin and PSTi8. α, HFrD mice vs drug treatment, β, chronic metformin vs chronic PSTi8 treatment. Glucose reduction experiment after acute administration of PSTi8 (i.p, 5 mg/kg) and metformin (p.o, 300 mg/kg) (**i**) C57BL/6J mice and (**j**) db/db mice. (**k**) Plasma concentration vs time profile of PSTi8 following intraperitoneal administration at dose of 5 mg/kg to db/db mice. Met: metformin *^,#^P < 0.05; **^,##^P < 0.01; ***^,###^P < 0.001. Error bar indicate mean ± s.e.m.

To compare the efficacy of PSTi8 with metformin, HFrD fed induced diabetic mice was choosen. In i.p GTT, AUC of chronic PSTi8 (31023.75 ± 872.22 mg/dL × min, P < 0.0001) (Fig. 3d) and acute PSTi8 (38997.5 ± 581.91 mg/dL × min, P < 0.0001) (Fig. 3c) showed more prominent improvement in glucose clearance compared to chronic metformin (36913.13 ± 891.89 mg/dL × min, P < 0.01) and acute metformin (44068.5 ± 3070.1 mg/dL × min, P < 0.01) respectively (Fig. 3a,c). Similarly, in ipITT AUC of chronic PSTi8 (14608.8 ± 392.41 mg/dL × min, P < 0.0001) (Fig. 3h), acute PSTi8 (18413.8 ± 480.21 mg/dL × min, P < 0.003) (Fig. 3f) showed more prominent improvement in glucose clearance compared to chronic metformin (16417.5 ± 1222.35 mg/dL × min, P < 0.0001) and acute metformin (19828.5 ± 1004.11 mg/dL × min) respectively. This is due to the increase in insulin sensitivity (Fig. 3e,g). PSTi8 did not show any decrease in glucose level in control C57Bl/6 mice (normoglycemic) compared to Metformin (Fig. 3i) which protects from hypoglycemia. However, in db/db mice (diabetic) PSTi8 showed decrease in gluconeogenesis and glucose level, which is similar to metformin (Fig. 3j). During PK study in db/db mice, plasma concentration of PSTi8 was observed upto 6 h (Fig. 3k).

### PSTi8 regulate secretion of bio-molecule, glycogen storage and hepatic gene expression

**Figure 4:**
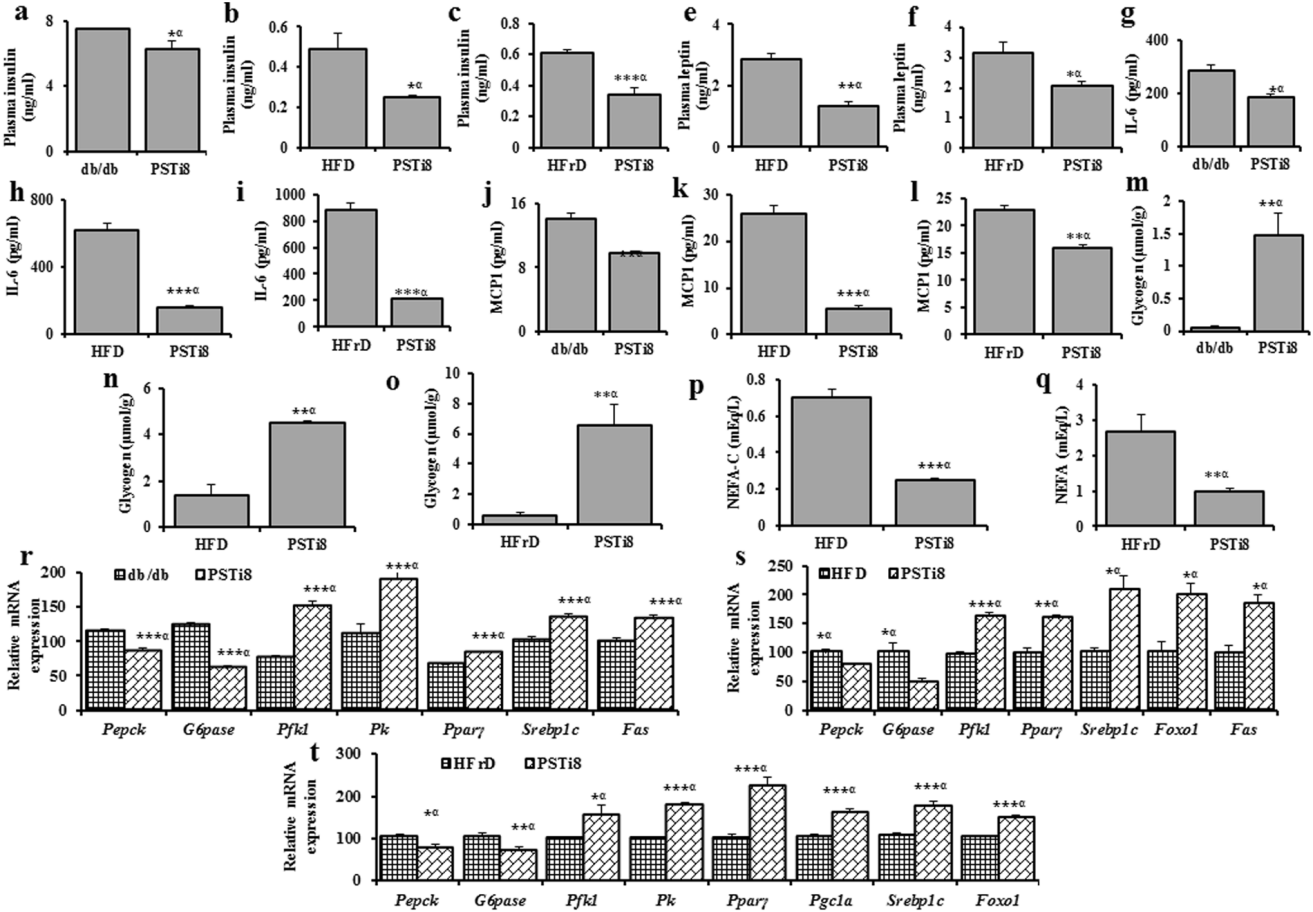
PSTi8 regulate secreation of biomolecule, glycogen storage and hepatic gene expression. Plasma concentrations of (**a**–**c**) insulin, (**d**–**f**) leptin, (**g**–**i**) IL-6, (**j**–**l**) MCP1, (**p**,**q**) NEFA-C and (**m**–**o**) liver glycogen in db/db, HFD, HFrD mice and PSTi8 treated db/db, HFD, HFrD mice. α, db/db, HFD, HFrD mice vs PSTi8 treated db/db, HFD, HFrD mice. (**r**–**t**) Effect of PSTi8 treatment on db/db, HFD and HFrD mice for the expression of glucose and lipid metabolic genes, (*Pepck*, *G6pase*, *Pfk1*, *Pk*, *Pparγ*, *Srebp-1c* and *Fas*) for db/db, (*Pepck*, *G6pase*, *Pfk1*, *Pparγ*, *Srebp-1c*, *FoxO1* and *Fas*) for HFD, (*Pepck*, *G6pase*, *Pfk1*, *Pk*, *Pparγ*, *Pgc1α*, *Srebp-1c* and *FoxO1*) for HFrD in liver tissue. α, db/db vs db/db + PSTi8, HFD vs HFD + PSTi8, HFrD vs HFrD + PSTi8. *P < 0.05; **P < 0.01; ***P < 0.001. Error bar indicate mean ± s.e.m.

The increase in insulin sensitivity, on PSTi8 treatment in HFD, HFrD and db/db mice may be due to inhibition of PST associated inflammation. PST induced higher level of circulating insulin due to IR^13^ was significantly decreased by PSTi8 treatment in HFD, HFrD and db/db mice (Fig. 4a–c). Treatments of PSTi8 significantly decreased production of IL-6 (Fig. 4g–i) and monocyte chromoattractant protein-1 (MCP-1)(Fig. 4j–l) pro-inflammatory cytokine levels in blood and inhibit inflammation which is found to be higher in hyperinsulimia^19^. Higher level of insulin and IL-6 stimulate leptin secretion which is decreased by PSTi8 treatment (Fig. 4e,f). Hyperinulimia also lead to hyperlipidemia condition and increased level of NEFA which was reversed by PSTi8 treatment in HFD (Fig. 4p) and HFrD (Fig. 4q) but there is no significant decreased in NEFA level in db/db mice (hence data not provided). This decreased level of NEFA level is due to fatty acid consumption. Treatment of PSTi8 also stimulates glycogen storage in liver tissue of HFD, HFrD and db/db mice (Fig. 4m–o). The changes in circulating level of these bio molecules and glycogen storage was due to the increased expression of *Pparγ*, *Srebp-1c*, fatty acid synthase (*Fas*), *Pgc1α*, *pyruvate kinase* (*Pk*), phosphofructokinase-1 (*Pfk1*), *FoxO1* and suppressed level of *G6pase* and *Pepck* genes in HFD (Fig. 4s), HFrd (Fig. 4t) and db/db (Fig. 4r) mice.

### Effect of PSTi8 on insulin signaling

**Figure 5:**
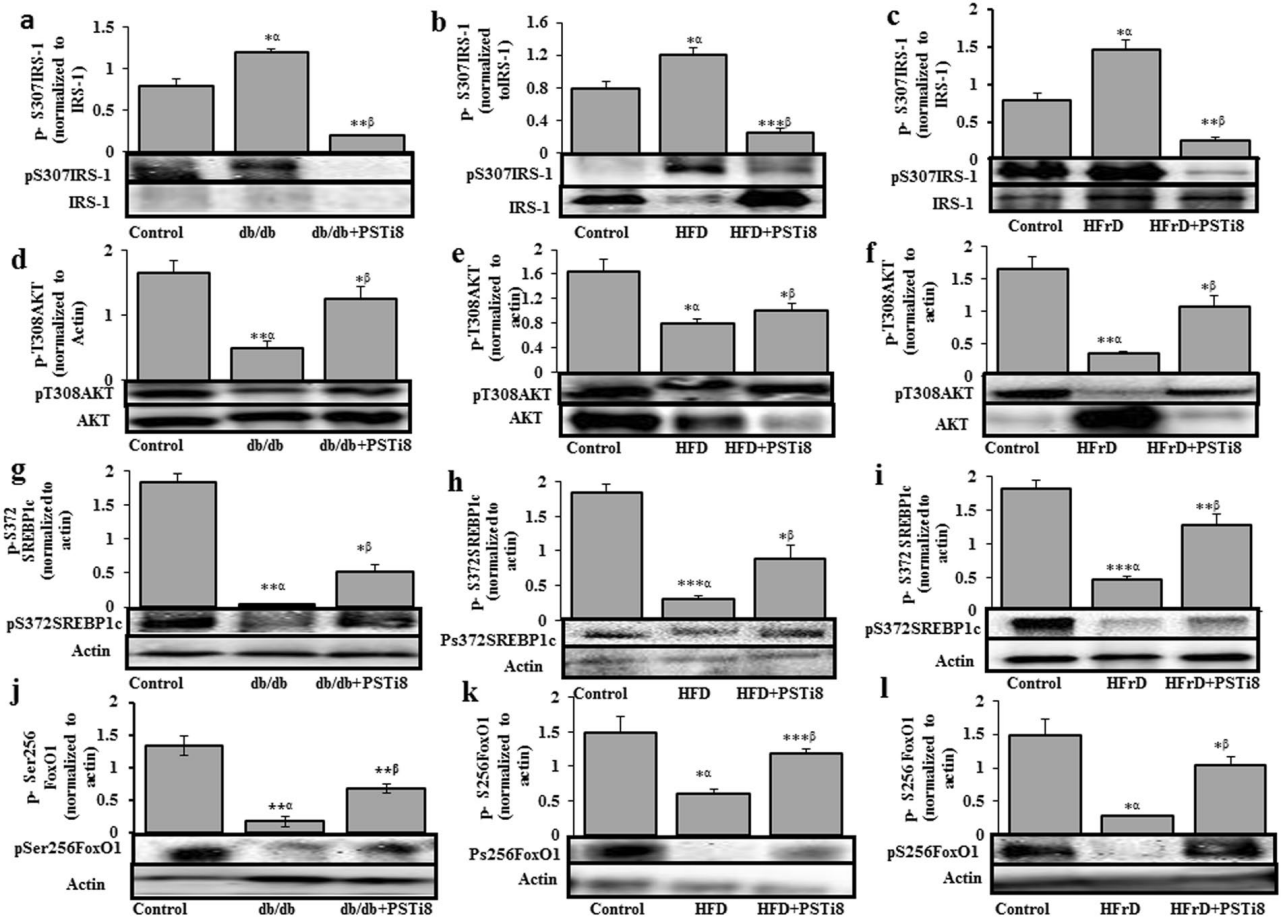
PSTi8 modulate insulin signaling. Western blot analysis of (**a**–**c**) phosphorylation of IRS-1 (p-S307IRS-1), (**d**–**f**) phosphorylation of Akt (p-T308AKT), (**g**–**i**) phosphorylation of Srebp-1c (p-S372SREBP1c) and (**j**–**l**) phosphorylation of FoxO1 (p-S256FoxO1) in liver tissues of db/db, HFD, HFrD mice and PSTi8 treated db/db, HFD, HFrD mice. α, control vs db/db, HFD, HFrD and β, db/db vs db/db + PSTi8, HFD vs HFD + PSTi8, HFrD vs HFrD + PSTi8. *P < 0.05; **P < 0.01; ***P < 0.001. Error bar indicate mean ± s.e.m.

PST have antagonistic effect on insulin signaling via PI3K activated Akt/FoxO1 and Srebp-1c pathway^4^. So we analyzed these pathways for inhibitory effect of PSTi8 on PST to activate insulin signaling pathway. Phosphorylation of IRS-1 at Ser307 by c-Jun N-terminal protein kinases and I kappa B kinase, results in inhibition of insulin signaling, a potential mechanism for IR^20^. The p-S307IRS-1 levels were decreased in PSTi8 treated db/db (Fig. 5a), HFD (Fig. 5b) and HFrD (Fig. 5c) mice. The decreased phosphorylation of IRS1 at (ser 307) recruit PI3 kinase which activates Akt by phosphorylation at (Thr308)^21^. The levels of p-T308Akt were increased in PSTi8 treated db/db (Fig. 5d), HFD (Fig. 5e) and HFrD mice (Fig. 5f). Activated Akt phosphorylates FoxO1 at (ser256) and inhibits its activity which results in inhibition of gluconeogenesis gene expression^4^. The levels of p-S256FoxO1 were found to be high in PSTi8 treated db/db (Fig. 5j), HFD (Fig. 5k) and HFrD mice (Fig. 5l). Besides activating Akt, PI3kinase also stimulate mRNA expression of Srebp-1c. The mRNA expression of Srebp-1c gene was found increased in PSTi8 treated db/db (Fig. 4r), HFD (Fig. 4s) and HFrD (Fig. 4t) mice. The active form of Srebp-1c is found in golgi apparatus by cleavage of inactive Srebp-1 by two protease sit1 and site2 proteases. Upon stimulus activated Srebp-1c enter nucleus and activates all lipiogenic genes in liver. But studies have shown that AMPK interacts with and directly phosphorylates Srebp-1c at (Ser372) which inhibits proteolytic cleavage of Srebp-1c and suppress lipogensis^22^. Treatment of PSTi8 stimulated phosphorylation of srebp1c at (ser372) in db/db (Fig. 5g), HFD (Fig. 5h) and HFrD mice (Fig. 5i). This process leads to reduction of lipid synthesis and accumulation in the liver. FoxO1 mediates signaling via pathway involving insulin-like growth factor receptor1, PI3K and Akt. It is inactivated through phosphorylation by Akt at (Ser256) which results in inhibition of gluconeogenesis gene expression. The levels of (p-S256) FoxO1 were found to be high in PSTi8 treated mice when compared to db/db (Fig. 5j), HFD (Fig. 5k) and HFrD mice (Fig. 5l).

### PSTi8 competes with PST on GRP78 receptor binding

**Figure 6:**
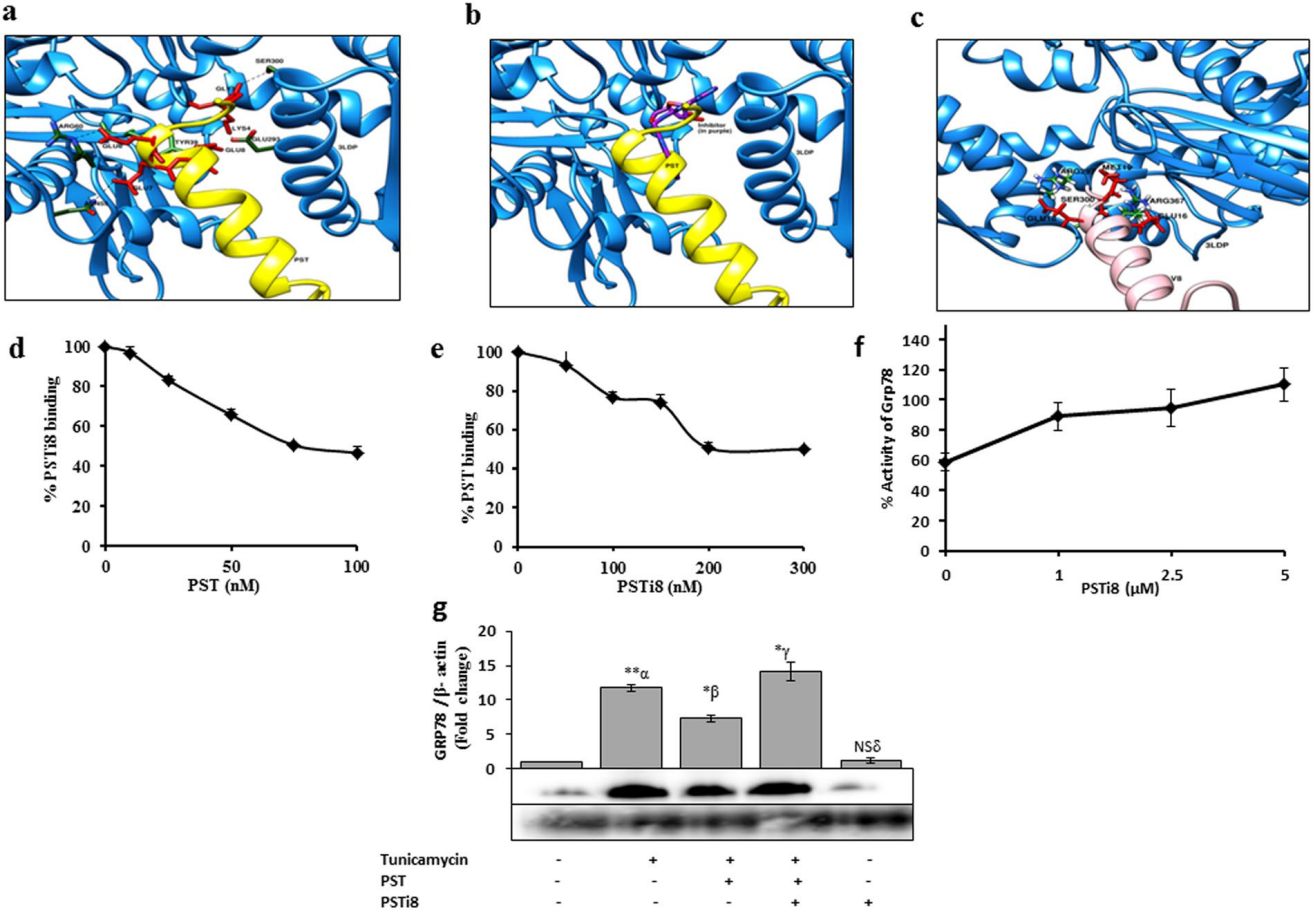
PSTi8 competes with PST on GRP78 receptor binding. Molecular docking (**a**) PST (yellow) is docked in the active site of human GRP78 (blue): the residues of PST in red are showing hydrogen bond with the residues of human GRP78 (green). (**b**) The superimposed image of PSTi8 (purple) and PST in the active site of human GRP78. (**c**) PSTi8 is docked in the active site of human GRP78: the residues of PSTi8 in red are showing hydrogen bond with the residues of human GRP78. (**d**) Competitive binding between different concentrations of PST and Sulphorhodamine labelled PSTi8 (150 nM) in HepG2 cells. (**e**) Competitive binding between different concentrations of PSTi8 and Sulphorhodamine labelled PST (25 nM) in HepG2 cells. (**f**) GRP78 ATPase activity in presences of PST (1 µM) with different dose of PSTi8 (0, 1, 2.5, 5 µM). Western blot analysis (**g**) Effect of PSTi8 (800 nM) on PST (100 nM) inhibited tunicamycin (5 mg/ml) stimulated GRP78 expression in HepG2 cells. α, control vs tunicamycin; β, Tunicamycin vs Tunca. + PST; γ, Tunica. PST vs Tunica. +PST + PSTi8 and δ, control vs PSTi8. *P < 0.05; **P < 0.01; ***P < 0.001, NS, Non-significant. Error bar indicate mean ± s.e.m.

To determine whether PSTi8 compete with PST on GRP78 receptor binding and regulate gluconeogenesis linked signaling in liver as seen in previous data. Circular Dichroism (CD) studies were performed to determine secondary structures of PST and PST inhibitors. The observed secondary structure (α-helix and β-sheet) of PSTi8 in aqueous buffer is close with PST (Supplementary Fig. 1 and Supplementary Table 1). Due to similar secondary structure, we have examined binding interaction of PST and PSTi8 with GRP78 protein. For this we have performed protein–protein docking studies using the GRAMM-X docking server. PST & PSTi8 were docked to the active site of human GRP78 at the same place as the hypothetical bound inhibitor was interacting. PST and PSTi8 involved in making hydrogen bonds with Glu293, Ser300 those are same as GRP78 inhibitor (Fig. 6a–c). PSTi8 also makes few more hydrogen bonds with Tyr39, Asn55 and Arg60. To validate further competitive nature of PSTi8 with PST on GRP78 receptor binding, we performed competitive binding with sulphorhodamine tagged PST and PSTi8. PST (100 nM) was found to inhibit >50% binding of labelled PSTi8 (150 nM) (Fig. 6d) and PSTi8 (300 nM) was found to inhibit >50% binding of labelled PST (25 nM) (Fig. 6e). From competitive binding experiments, it was found that the PSTi8 is a potent PST inhibitor. Further to evaluate competitive nature of PSTi8, we performed ATPase activity and protein expression of GRP78. ATPase activity of GRP78 is required for the downstream processing of the molecule which regulate G6pase gene expression^11^. PSTi8 competed with PST in dose dependent manner to bind on GRP78 receptor. It enhances ATPase activity of GRP78 ∼50% at 5 µM, inhibited by 1 µM of PST (Fig. 6f). PSTi8 directly did not effect GRP78 expression. PST (100 nM) inhibits protein expression of GRP78 in tunicamycin stimulated condition in HepG2 cells^11^ which is significantly inhibited by PSTi8 (800 nM) and enhances tunicamycin effect on stimulation of GRP78 protein expression (Fig. 6g).

## Discussion

The current study demonstrated *in*-*vitro* & *in*-*vivo* anti-diabetic activity of PSTi8 as a PST inhibitory peptide. The current finding is that, PSTi8 improve insulin effect on glucose uptake in hepatocytes, adipocytes and IR cells by regulating glucose and fatty acid metabolism gene (*in*-*vitro*). PSTi8 increases glucose uptake by increasing GLUT4 translocation on cell surface and suppress hepatic glucose production through the regulation of gluconeogenic genes. PSTi8 inhibit PST binding on GRP78 receptor and modulates its activity and expression. PSTi8 improves glucose homeostasis and insulin sensitivity by reducing the level of pro-inflammatory cytokines and control glucose and fatty acid metabolic gene expression in HFD, HFrD and db/db diabetic mice through the regulation of Akt-FoxO1 and Srebp-1c pathway. These results suggest that PSTi8 ameliorate glucose and lipid homeostasis.

Insulin resistance is a major complication associated with diabetes^23^. Our *in*-*vitro* data suggest that PST inhibited glucose uptake in insulin stimulated HepG2 (Fig. 1a) and 3T3L1 (Fig. 1c) cells was reversed by PSTi8 due to anti-PST effect. The increase in glucose uptake was coupled with increases in glycolysis^24^ (Fig. 1e). Beside PST induce IR effect, PSTi8 also attenuate high glucose induced IR in HepG2 cells^15^ (Fig. 1b) and in un-induced condition (Fig. 1a) by stimulating glucose uptake. Excessive hepatic glucose production is associated with hyperglycemia^25^ and leads to diabetes. The stimulatory effect of PST and glucagon on hepatic glucose production^4,26^ is inhibited by PSTi8 (Fig. 1d). The inhibitory effects of PSTi8 on gluconeogenesis occur due to inhibition of *Pepck*, *Pc* and *G6pase* gene expression (Fig. 1e,i). PST does not show anti-insulin effect on glucose uptake in muscle cells^4^ but PSTi8 stimulate GLUT4 translocation (Fig. 1i) which leads to more glucose uptake in L6 cells (Fig. 1h). PSTi8 also modulate PST effect on insulin in 3T3L1 cells (adipocytes)^1,27^. Adipocytes store and distributes energy in the form of fat which serves fuel throughout the body^28^. The increase in lipogensis and adipogensis is required for the differentiation of preadipocytes to adipocytes which is stimulated by insulin via enhancing *Pparγ*, *Scd1*, FoxO1 and Pgc1α gene expression^29–31^. The increase in lipid content in adipocytes is coupled with increase in *Pparβ* gene expression, this shifts metabolism towards fat from glucose^32^ and increases *Ucp2* gene expression^33^. This effect of insulin on adipocytes was masked by PST and disrupt lipid metabolism, which is rescued by PSTi8 treatment (Fig. 1g).

PSTi8 which shows significant *in*-*vitro* anti-diabetic activity was further utilized in the *in*-*vivo* experiments. As per the previous studies, PST deficiency in normal chow diet fed *Chga* KO mice and HFD fed *Chga* KO mice showed more insulin sensitivity than wild type mice and HFD fed wild type mice, respectively. PST supplementation causes IR in *Chga* KO mice by stimulating *Pepck* and *G6pase* mRNA abundance which causes rise in blood glucose levels^4^. PST supplementation causes inflammation and IR in KO-DIO mice. Obesity is mainly associated with IR in the presence of PST and in its absence obesity is dissociated with IR^13^. The level of PST was found increased in DIO (diet induced obesity) and db/db mice^13^ which causes development of insulin resistance. Previous study suggested that PST administration induce insulin resistance in *Chga* KO mice^4^. So we evaluated the anti-diabetic activity of PSTi8 in three different mice models, HFD, HFrD-fed and db/db mice^34–37^. GTT, ITT and PTT were performed in these models, showed high basal blood glucose, high blood glucose-time profiles and AUC^37^. Treatment of PSTi8 significantly decreased these index of insulin resistance associated with PST and enhance glucose clearance, insulin sensitivity and suppress gluconeogenesis. The effect of PSTi8 was found more in chronic compare to acute treatment in HFD (Fig. 2c,d,i,j,o,p), HFrD (Fig. 2e,f,k,l,q,r) and db/db mice (Fig. 2a,b,g,h,m,n). So chronic treatment of PSTi8 show better efficacy at low dose as compare to acute dose. In normoglycemic mice, PSTi8 did not cause further decrease in blood glucose level (Fig. 3i) but in diabetic db/db mice it reduced blood glucose level (Fig. 3j). Unlike metformin PSTi8 does not induce hypoglycemia in wild type mice (Fig. 3i) and works when there is glucose challenge to maintain glucose homeostasis (Fig. 3j). The glucose clearance and insulin sensitivity of chronic and acute PSTi8 treatment was more than chronic (Fig. 3b,d) and acute (Fig. 3f,h) metformin treatment. So it shows more promising effect in term of efficacy as compared to metformin^38^.

Inflammation is the major cause of insulin resistance which occur due to overproduction of IL-6 and MCP-1 (pro-inflammatory) cytokines^39^. The increased MCP-1 level leads to infiltration of monocytes into peripheral tissue to replenish macrophages, which causes overproduction IL-6^40^. The level of these inflammatory cytokines was found decreased in *Chga* KO-DIO mice due to absence of PST^13,41^. This indicate inflammatory role of PST in inducing IL-6 and MCP-1 production and insulin resistance. The effect of PST on IL-6 and MCP-1 production was impaired by PSTi8 treatment in HFD (Fig. 4h,k), HFrD (Fig. 4i,l) and db/db (Fig. 4g,j) mice. The elevated insulin level in IR was found in DIO, db/db and PST administered *Chga* KO DIO and decreased in *Chga* KO mice in compared to WT mice^4,42,43^. Since PST presences causes IR which directly enhances the insulin secretion, is suppressed by PSTi8 administration in HFD, HFrD and db/db mice (Fig. 4a–c). Both higher level of insulin and IL-6 stimulate leptin secretion from adipose tissue and causes inflammation in HFD, HFrD and db/db mice^44^. *Chga* KO mice has higher level of leptin due to prolonged effect of insulin on Srebp-1c gene expression, causes obesity which was reversed by PST^13^. Administration of PSTi8 significantly lower leptin secretion (Fig. 4e,f) may be the cause of decrease in obesity^45^. In indirect calorimeter data, we have found decreased trend in basal metabolic rate (BMR) and resting metabolic rate (RMR) in PSTi8 treated HFD, HFrD and db/db mice (Supplementary Fig. 2). Glycogen is a major energy storage molecule in liver and maintains glucose homeostasis in fasting stage. Lower level of glycogen causes insulin resistance by inhibiting Akt stimulated insulin pathway and lead to steatosis^46^. PST administration inhibit glycogen storage in liver and stimulate gluconeogenesis in *Chga* KO-DIO mice^13^. Treatment of PSTi8 inhibit PST effect and stimulate hepatic glycogen storage (Fig. 4m–o) and inhibit hepatic glucose release as shown in PTT (Fig. 2m,o and q). The PSTi8 shows these effects by inhibiting gluconeogenic gene expression (G6pase, Pepck) (Fig. 4r–t) and causes overexpression of ppar gamma^47^ (Fig. 4r–t). Non esterified fatty acid (NEFA) released by lipolysis of triglyceride and promote insulin resistance. Higher level of NEFA was detected in HFD/HFrD mice due to development of hyperlipidemia^48^. In *Chga* KO mice due to lack PST NEFA level got decreased in compared to WT^4^, means PST stimulate NEFA production as seen in DIO. This effect of PST was significantly suppressed by PSTi8 treatment in diabetic mice (Fig. 4p,q).

**Figure 7:**
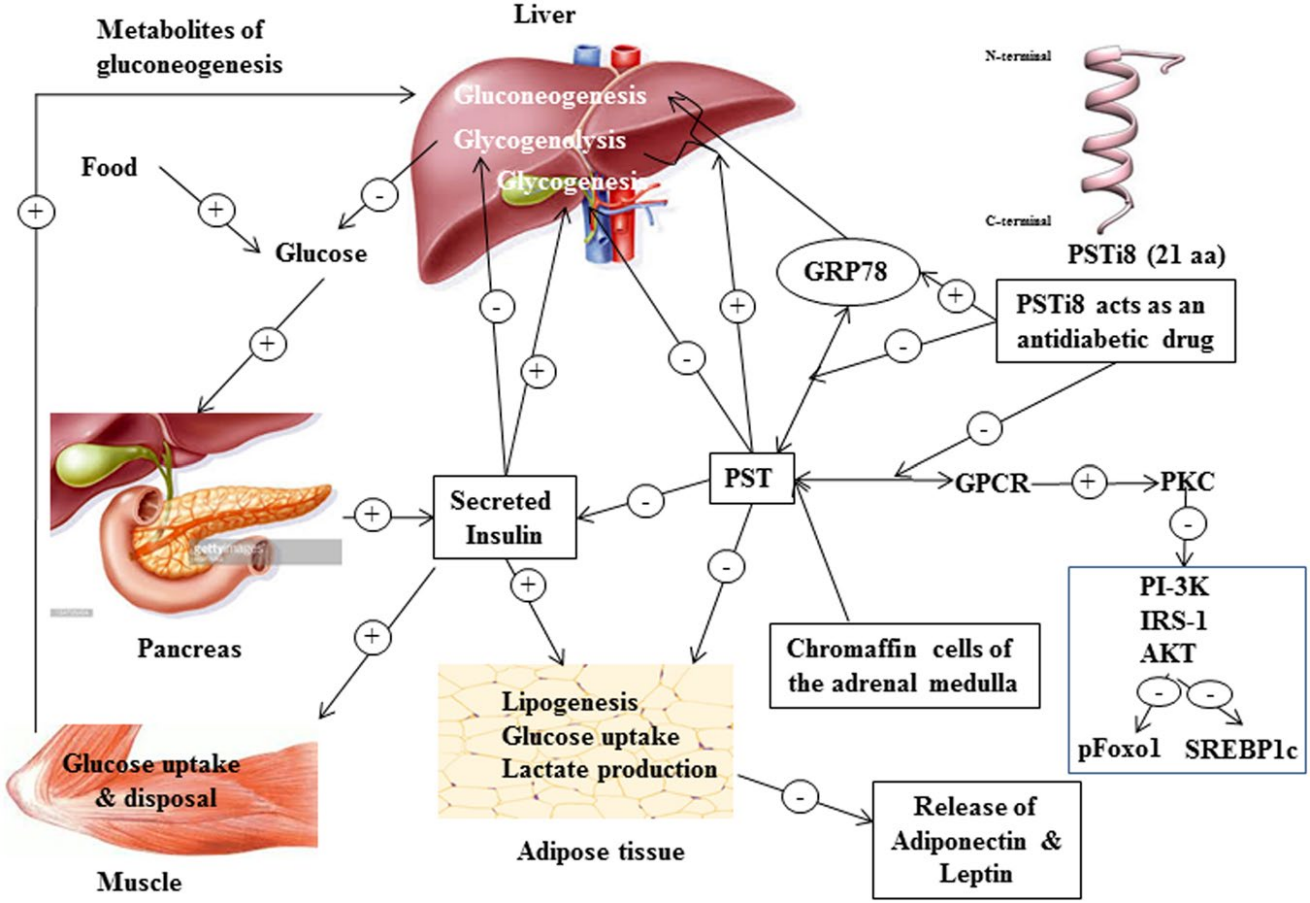
Mechanism of action of PSTi8 towards glucose and energy homeostasis. PSTi8 pre-occupy the GRP78 receptor where PST binds. PST regulation of glucose homeostasis in different tissues which is inhibited by PSTi8 in metabolic syndrome and type-2 diabetes. Schematic diagram showing the inhibitory effect of PSTi8 on PST at PST receptors in the liver. GPCR, G protein-coupled receptor; PKC, protein kinase c; PI-3K, phosphatidylinositol 3-kinase; pFoxO1, phosphorylated FoxO1; Srebp-1c, Sterol regulatory element-binding protein 1c; Akt, protein kinase B; PIP3, Phosphatidylinositol (3,4,5)-trisphosphate; IRS1, Insulin receptor substrate 1; GTP, Guanosine triphosphate; cGMP, Cyclic guanosine monophosphate; NOS, Nitric oxide synthase.

PST is known to regulate IR either by interacting with GRP78 receptor (glucose response protein), located in ER (endoplasmic reticulum)^11^ or by binding with G protein coupled receptor. GRP78 expression decreased in diet induced and db/db mice which stimulate G6pase expression^49^. The decrease in G6pase expression was due to higher circulating level of PST which suppress GRP78 expression and its ATPase activity over the ER stress (during high calorie diet intake^11^). Administration of PSTi8 suppress PST action, stimulate GRP78 activity (Fig. 6f) by competitive inhibition (Fig. 6d,e). PSTi8 does not regulate GRP78 expression but rescues the inhibitory effect of PST and tunicamycin on GRP78 expression (Fig. 6g). The administration of PST in PST-deficient DIO mice or higher circulating level in DIO hyperglycemia and db/db mice inhibit insulin mediated PI3kinase activated signaling pathway^4,13^. PST on binding GPCR inhibit insulin signaling by activating conventional protein kinase C (cPKC) dependent inactivation of PI-3 kinase which regulate Akt/FoxO1 and Srebp-1c pathway^4^. Activated cPKC causes phosphorylation of IRS-1 at (Ser307) which inhibit the binding of p85 regulatory subunit of PI3kinase and lead to its inactivation. Inactivated PI3kianse could not further activate Akt and other downstream transcription factors such as FoxO1 and Srebp-1c which leads to insulin resistance^50^. The activated FoxO1 (unphosphorylated) in the nucleus bind to the promoter of *G6pase* and *Pepck* gene and stimulate their expression which enhances gluconeogenesis^51^. On insulin treatment activation of Akt via PI3kinase stimulate phosphorylation of FoxO1 at (Ser256) position cause exclusion from the nucleus and inhibit its transcription activity^4^. In our data, treatment of PSTi8 decreases phosphorylation of IRS-1 at (Ser307) (Fig. 5a–c) and enhance insulin signaling through the recruitment of PI3kinase which causes the activation of Akt through phosphorylation at (Thr308) (Fig. 5d–f) and stimulate nuclear exclusion through phosphorylation of FoxO1 (Ser256) (Fig. 5j–l) and suppress gluconeogenic gene (*G6pase*, *Pepck*) expression^4^ (Fig. 4r–t). Activated Akt stimulate glycogen synthesis (Fig. 4m–o) and glucose clearance in PSTi8 treated mice as seen in GTT (Fig. 2a,c,e). Activation of Akt also stimulates mRNA expression of *Srebp-1c* gene (Fig. 4r–t) which causes lipogensis^52^. But in PSTi8 treated mice the phosphorylation of Srepb-1c at (ser372) increases (Fig. 5g–i) which inhibit proteolytic cleavage of precursor form into mature Srebp1c form and causes inhibition in lipogensis^22^. The decreased lipogensis is not coupled with increased fatty acid synthesis gene *Fas* (Fig. 4r–t) which was due to increased expression of *Pparα* and *Cpt1a* (Fig. 1f) in hepatocytes which would in turn lead to enhanced β-oxidation^53^, a pathway for fatty acid catabolism. It concludes that PSTi8 ameliorate glucose and lipid metabolism through PI-3k/akt mediated modulation of FoxO1 and Srebp-1c in diabetic mice. The effects of PST & PSTi8 actions are schematically represented in Fig. 7.

## Conclusions

PSTi8 provides a potential therapeutic agent for the treatment of IR and diabetes. Our studies confirms that PSTi8 possess antidiabetic activity in both *in*-*vitro* and *in*-*vivo*. Our data reported that PSTi8 treatment improved the glucose homeostasis in IR mice, characterized by elevated glucose clearance, enhanced glycogenesis, enhanced glycolysis and reduced gluconeogenesis. PSTi8 enhances insulin sensitivity and maintains euglycemia in IR mice, by reversing the effect of PST and reducing inflammatory cytokines and lipogensis. In liver PSTi8 enhances insulin sensitivity by activating IRS1/2-PI3K-Akt-FoxO1 (Srebp-1c) signaling pathways. Based on our results an Indian patent is filed (No. 201611010438). Further investigation is required to determine whether PSTi8 improve glucose and lipid homeostasis in muscle and adipose tissue. In conclusion, discovery of PSTi8 as PST inhibitor, that remarkably improves metabolic health in diabetes provides a promising therapeutic tool for the treatment of genetically & lifestyle induced diabetes.

## Methods

To check the anti PST activity of PSTi8 and to explore it’s evaluation as ant-diabetic therapeutic peptide, we have designed number of experiments such as glucose uptake, hepatic glucose production and GLUT4 translocation. Further evaluation of PSTi8 as glucose homeostasis regulator, GTT, ITT and PTT were performed to investigate glucose clearance, insulin sensitivity and gluconeogenesis respectively in IR models. To track the signaling pathways we performed rt-qpcr and western blot. In slico protein- protein docking and GRP78 ATPase assay was performed to find the competitive nature of PSTi8 and for anti-inflammatory effect ELISA was done.

### Synthetic peptides

Wild type human PST (PEGKGEQEHSQQKEEEEEMAVVPQGLFRG-amide) and PST inhibitor series, including PSTi8 (PEGKGEQEHSQQKEEEEEMAV-amide) were designed by addition/deletion/modification of amino acids from both the terminal of PST sequence. Peptides were synthesized and purified from Life Tein LLC, New Jersey, USA. The sequences of all PST inhibitory peptides are available in Supplementary Table 2.

### Reagents and kits

Dexamethasone, 3-Isobutyl-1-methylxanthine (IBMX), Insulin, 2′,7′“Dichlorofluorescin diacetate (DCFDA), TRIzol, Glycogen, STZ, Anthrone, Bradford reagent, FBS, Trypsin-EDTA, DMEM were purchased from Sigma, USA. Antibiotic-antimycotic solution, Amplex red glucose/glucose oxidase assay kit, high capacity RNA to cDNA Reverse Transcriptase kit were purchased from Applied Biosystems, USA. 2xSYBR green Premix ExTaq was purchased from Takara Bio, Japan. Primers for RT-qPCR were purchased from Eurofins Scientific, Germany. Tritium radiolabeled deoxy-D-Glucose,2,1-2-3H[N](2-DG) was purchased from American Radiolabeled Chemicals Inc. USA. High fat diet (60%, Cat# D12492) and high fructose diet (60%, Cat# D00111301) were obtained from Research Diets, Inc, USA. All antibodies were purchased from Cell Signaling Technology(Beverly, MA) and Santa Cruz Biotechnology(Santa Cruz, CA).

### 2-Deoxyglucose (2-DG) uptake assay

2-DG uptake assay was performed in HepG2, IR-HepG2, 3T3L1 and L6 cells^4^. After 24 hr of seeding, cells were treated with or without PST (25 nM) and/or PSTi8 (150 nM) for 20 hr. For IR, HepG2 cells treated with 30 mM glucose [prepared by supplementing D-glucose in HGDMEM (cat no. D5648) medium] for 48 hr^15^. Cells were serum starved for 4 hr along with PST and/or PSTi8. Then the cells were incubated with insulin (100 nM) for 30 min in 0.5 mL of warm (37 °C) krebs ringer hepes (KRH) buffer. Glucose uptake was initiated by the addition of 0.3 mL of KRH buffer containing 10 µM 2-DG (0.5 µCi/mL 2-[3 H] DG) to each well. It was terminated after 5 min and the cells were solubilized with 0.05 M NaOH, and radioactivity was measured by beta-counter (Beckman Coulter, USA) and normalized by protein content to measure fold change in glucose uptake among treated and untreated (control)^19–21^.

### Glucose production in HepG2 cells

HepG2 cells were incubated for 20 hr in a complete LGDMEM medium with positive control (Glucagon, 6 nM) and test samples (PSTi8, 150 nM and metformin, 100 µM). After serum starvation, glucose release was performed for 4 hr by incubating with glucose production media and release was measured by Amplex red assay kit. Glucose release was normalized to protein content^16^.

### GLUT4 translocation

L6-GLUT4myc cells were seeded in 24 well plate and after reached confluence, differentiated in differentiating media (2% FBS + αMEM) for 4–6 days and an assay was carried out with 30 min exposure of insulin (200 nM), and PSTi8 (150 nM). After treatment cells were washed with PBS and fixed with 3% paraformaldehyde in PBS for 3 min at room temperature. The fixative solution neutralized by adding 1% glycine for 10 min at 4 °C and blocked with blocking solution (10% goat serum and 3% BSA in PBS) for 30 minutes. Primary antibody (anti-c myc C3956) then added in a dilution of 1:500 for 45 minutes at 4 °C, removed and washed with PBS and incubates with peroxidase conjugated rabbit anti-mouse IgG (1:1000). After 30 minutes of incubation, cells were washed extensively with PBS before adding 1 ml of OPD in each well. The colorimetric reaction was stopped by addition of 0.25 ml of 3 N HCl at room temperature. The supernatant collected and read the absorbance at 492 nm by using spectrophotometer^54,55^.

### Animal model development and treatment

All animal experiments were performed according to protocols approved by Institutional Animal Ethics Committee (IAEC approval no. IAEC/2012/31-A), CSIR-Central Drug Research Institute (CSIR-CDRI, India). Male C57BL/6 mice (8–10 weeks, 15–20 g) and male db/db mice (10–12 weeks, 45–50 g) were obtained from Laboratory of Animal Division, CSIR-CDRI. For diet induced obesity/diabetes, feeding was started at the age of two months of C57BL/6 mice with 60% HFD and 60% HFrD, continued for 10–12 weeks with a dose of STZ single injection (100 mg/kg i.p) on the 4^th^ week of feeding, which did not affect insulin level in normal C57BL/6 mice but stimulate blood glucose level in fat/fructose fed mice^34,36^. After successful of model development diabetic mice divided in two groups, vehicle control group receive saline and treated group receive PSTi8. After acute (5 mg/kg) and chronic (2 mg/kg) treatment of PSTi8 for 10 days, mice were fasted for 6 hr before experiments and then sacrificed to collect plasma and tissues, which were snap frozen in liquid nitrogen.

### Glucose, insulin and pyruvate tolerance tests

i.p GTT, i.p ITT and i.p PTT were carried out in 6 hr fasted mice. The blood glucose level was measured from tail tip using Contour TS blood glucose meter. The AUC is calculated using GraphPad Prism 5.0 software^4,13^.

### Measurement of insulin, lipid and adipokine levels in circulation

Plasma insulin and leptin level were assayed using enzyme-linked immunosorbent assay (ELISA) kit (CrystalChem, USA). Plasma NEFA-C was assayed using NEFA-C kit, Wako Diagnostics(VA). Plasma IL-6 and MCP-1 were estimated using ELISA kit, RayBiotech(USA).

### Liver glycogen content

Liver tissue was dissolved in hot KOH (30%) then glycogen was precipitated in ethanol (at 4 °C) and centrifuged at 5000 rpm for 12 min. The pellet was redissolved in 5 mL water, and 1 mL was mixed with 3 mL of Anthrone reagent (0.2% Anthrone in concentrated H_2_SO_4_) to determine the concentration of glycogen at 620 nm by comparison with standard glycogen^4^.

### Competitive binding

Sulphorhodamine B labelled PST and PSTi8 were synthesized and purified from Genpro Biotech, India. In HepG2 cells, the competitive binding assay of PST and PSTi8 were performed and the labelled PST and/or PSTi8 concentrations were measured at 568 nm and 584 nm, respectively^56^.

### Molecular docking (MD)

The structure of PST and PSTi8 were modelled using I-Tasser server. A MD simulation with the help of Gromacs 4.5.7 was performed to obtain an equilibrated structure. The structure obtained after 10 ns of MD simulation was taken further for performing docking experiments with structure of human GRP78. The Crystal structure of human GRP78 ATPase domain in complex with a small molecule inhibitor was obtained from the protein data bank (PDB ID: 3LDP)^57^.

### Circular dichroism (CD)

The CD spectra of the peptides were recorded on a Jasco J-170 spectropolarimeter in PBS(pH 7.4). The samples were scanned at room temperature with the help of a capped quartz cuvette of 0.2 cm path length at a wavelength range 250–190 nm. An average of 4–6 scans was taken for each sample^58^.

### Real time qPCR (RT-qPCR)

Total RNA was extracted from cells and mice tissues using TRIzol reagent. RNA was transcribed into cDNA using High Capacity RNA to cDNA Reverse Transcriptase kit, analyzed, and amplified using reverse transcriptase PCR using SureCycler 8800(Agilent Technologies, USA). RT-qPCR was performed on Light Cycler 480 II (Roche Diagnostics) with SYBR Green fluorescent label. Cycle threshold (Ct) values were used to calculate the amount of amplified PCR product^59^. The list of primer sequence is listed in supplementary Table 3.

### Western blotting

Frozen tissues and cells were homogenized in liquid nitrogen and lysed in lysis buffer containing phosphatase and protease inhibitors. After running equal amounts of protein on 10% SDS-PAGE, gels were transferred to polyvinylidene difluoride (PVDF) membrane. Blocking of nonspecific binding proteins on membrane was performed using 5% skim milk powder in TBST (2 hr, RT). Membranes were then probed with primary antibodies, rabbit anti-phospho IRS-1 (Ser307) (#2381), rabbit anti-Insulin receptor substrate (IRS-1) (#2382), rabbit anti-phospho AKT (Thr308) (#4056), rabbit anti-AKT (pan) (#4691), rabbit anti-phospho FoXO1 (Ser256) (#9461), rabbit anti-phospho Srebp1c (Ser372) (9874 S), GRP78 Bip (C50B12) (#3177) and rabbit anti-β actin (#4967) purchased from Cell Signaling- Technology (Beverely, MA) and in [1:1000 dilution of 5% BSA in TBST, except β-actin used 1:3000] overnight at 4 °C. After 4–5 washes with TBST, the membranes were then incubated with HRP conjugated goat anti-rabbit secondary IgGs (#7074), and horse anti-mouse IgG (#7076) at 1:3000 dilution for 2 h at RT, washed and then visualized by ECL detection kit (Millipore, USA). The signals were normalized with their respective gene or β-actin by using National Institute of Health (NIH) Image J software^59^.

### GRP78’s ATPase enzymatic activity

To determine that PSTi8 inhibit PST inhibitory action on GRP78 ATPase activity, we perform Concentration-dependent spectrophotometric assays using recombinant human GRP78 protein (catalog # ab78432, Abcam Inc., Cambridge, MA). PSTi8 was co-incubated with PST (1 µM) at different doses (1, 2.5 and 5 µM) in 50 µl of assay buffer (20 mM Tris, pH 7.5, 50 mM KCl, 1.5 mM MgCl_2_) containing GRP78 protein (0.25 µM). The reaction was started by adding 100 µM ATP and incubated at 37 °C for 30 min. On ATP hydrolysis free phosphate (Pi) was measured by a Malachite green-phosphate assay (catalog #10009325, Cayman)^11^.

### Indirect calorimetry

Metabolic measurement and analysis were performed by indirect calorimetry using Oxymax Comprehensive Laboratory Animal Monitoring System (CLAMS) (Columbus Instruments, Columbus, USA).

### *In*-*vivo* pharmacokinetic (PK) study

PSTi8 (5 mg/kg) was administered (i.p) in db/db mice for PK study. After dosing, blood samples were collected at different time points. Plasma samples (100 µL) were processed using the solid phase extraction (SPE). The 100 µL of eluted SPE samples were injected into the liquid chromatography tandem mass spectrometry (LC-MS/MS) (API 4000QTRAP, ABSciex, Canada) for analysis. The PK profile and parameters were evaluated by non-compartmental model approach using Pheonix 6.3 WinNonlin(Pharsight Corporation, USA).

### Data analysis

Results are expressed as mean ± SEM. All the data were analyzed using GraphPad Prism 5.0 software. Statistical analyses were performed using Student *t* tests, one- and two- way ANOVA followed by Bonferroni posttest wherever appropriate. A value of p < 0.05 was considered to be statistically significant.

## Electronic supplementary material

Supplementary File
