# Supplementary material for: Discovery of pancreastatin inhibitor PSTi8 for the treatment of insulin resistance and diabetes: studies in rodent models of diabetes mellitus
Source: Sci Rep. 2018 Jun 7;8:8715. doi: 10.1038/s41598-018-27018-8 (PMC5992141; doi:10.1038/s41598-018-27018-8)

Discovery of pancreastatin inhibitor PSTi8 for the treatment of insulin resistance and diabetes: studies in rodent models of diabetes mellitus

Zakir Hossain[1#], Guru R. Valicherla[1,3#], Anand P. Gupta[1#], Anees A. Syed[1], Mohammed Riyazuddin[1], Sharat Chandra[2], Mohammad I. Siddiqi[2], Jiaur R. Gayen[1,3*]

Supplementary Table 1: Peptide secondary structure analysis by circular dichroism using the K2D3 software

Peptide Name	Molecular Weight (kDa)	Alpha helix (%)	Beta sheet (%)	Anti-PST activity
S1/PST	3.28	8.82	16.54	
S2/PSTi8	2.427	9.07	16.52	+++
S3/PSTi7	2.624	12.06	15.41	+
S4/PSTi2	2.685	2.07	17.62	-

Supplementary Table 2. List of PST inhibitor peptides sequence

Name	Peptide Sequence
PSTi1	GKGEQEHSQQKEEEEEMAVVPQGLFRG, 27 aa, Amidation (C-Terminal)
PSTi2	GEQEHSQQKEEEEEMAVVPQGLFRG, 25 aa, Amidation (C-Terminal)
PSTi3	QEHSQQKEEEEEMAVVPQGLFRG, 23 aa, Amidation (C-Terminal)
PSTi4	HSQQKEEEEEMAVVPQGLFRG, 21 aa, Amidation (C-Terminal)
PSTi5	PEGKGEQEHSQQKEEEEEMAVVPQGLF, 27 aa, Amidation (C-Terminal)
PSTi6	PEGKGEQEHSQQKEEEEEMAVVPQG, 25 aa, Amidation (C-Terminal)
PSTi7	PEGKGEQEHSQQKEEEEEMAVVP, 23 aa, Amidation (C-Terminal)
PSTi8	PEGKGEQEHSQQKEEEEEMAV, 21 aa, Amidation (C-Terminal)
RI-PSTi7	pvvameeeeekqqsheqeGkGep, 23 aa, Amidation (C-Terminal)
RI-PSTi8	vameeeeekqqsheqeGkGep, 21 aa, Amidation (C-Terminal)

Supplementary Table 3. Primer sequence of related genes used for RT qPCR

Gene name	Forward primers	Reverse primers
hPfk1	GAAGTCAAACTGAATGTGTC	CCTCTTGTAGGCAGTAAGTC
hHk	CACCTGTGAGGTTGGACTCA	CCACCATCTCCACGTTCTTC
hPepck	AGATGGAGGAAGAGGGCATC	GTGAGAGCCAACCAGCAGTT
hG6pase	ACTGGTTCAACCTCGTCTT	CGAAAGATAGCGAGAGTAGA
hPparα	CCGCAATGGACCATGTAAC	CAGCTCTAGCATGGCCTTTT
hCpt1a	CACCTCCGTAGCTGACTCG	CGTGAACTGAAAGGCCACA
hSrebp1c	GTGGGCACTGAGGCAAAG	GACAGCAGTGCGCAGACTTA
hScd1	CCTAGAAGCTGAGAAACTGGTGA	ACATCATCAGCAAGCCAGGT
hGapdh	AGCCACATCGCTCAGACAC	AATACGACCAAATCCGTTGACT
hβactin	CTACGTCGCCCTGGACTTCGAGC	GATGGAGCCGCCGATCCACACGG
mUcp2	GTTCCTCTGTCTCGTCTTGC	GGCCTTGAAACCAACCA
mPgc1a	AGCCGTGACCACTGACAACGAG	CTGCATGGTTCTGAGTGCTAAG
mFoxo1	CTTCAAGGATAAGGGCGACA	GACAGATTGTGGCGAATTGA
mAkt1	TCGTGTGGCAGGATGTGTAT	ACCTGGTGTCAGTCTCAGAGG
mPparβ	TCCAGAAGAAGAACCGCAACA	GGATAGCGTTGTGCGACATG
mPepck	ATGTGTGGGCGATGACATT	AACCCGTTTTCTGGGTTGAT
mG6pase	CATGGGCGCAGCAGGTGTATACT	CAAGGTAGATCCGGGACAGACAG
mPfk1	GGAAGCCAAATGGGACTGT	CGCACTACCGATGATGGTC
mPk	GTGGAGGCTTCCTTCAAGTG	AGGTCGGTAGCGAGACAGAA
mPparγ	CAGGCCGAGAAGGAGAAGCT	GGCTCGCAGATCAGCAGACT
mSrebp1c	GGTTTTGAACGACATCGAAGA	CGGGAAGTCACTGTCTTGGT
mFas	CGGAAACTTCAGGAAATGTCC	TCAGAGACGTGTCACTCCTGG
mGapdh	GTGTCCGTCGTGGATCTGA	CCTGCTTCACCACCTTCTTG
mβactin	CCTCACCCTCCCAAAAGC	GTGGACTCAGGGCATGGA
hPyruvate carboxylase	GACTGTTCGGAACTTCAGCAT	GAGGCAGCAGCGGTAGAG

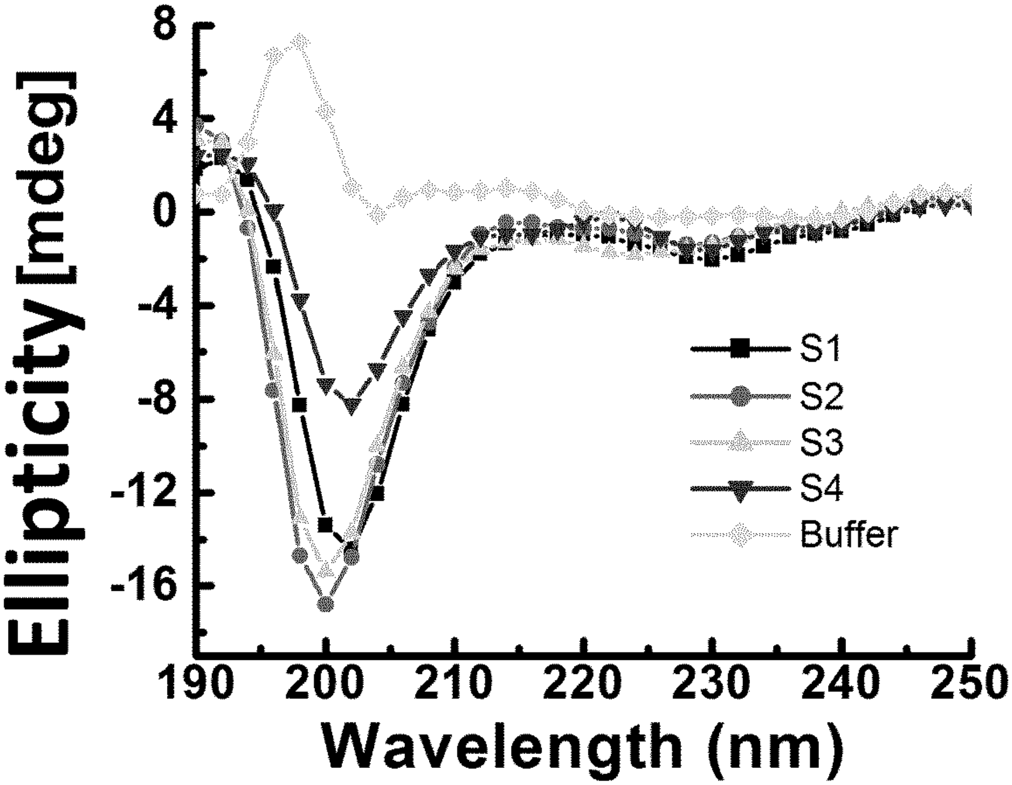

Supplementary Figure 1. Determination of secondary structures of PST and PST inhibitors in aqueous environment using circular dichroism (CD). S1: PST, S2: PSTi8, S3: PSTi7, S4: PSTi2.

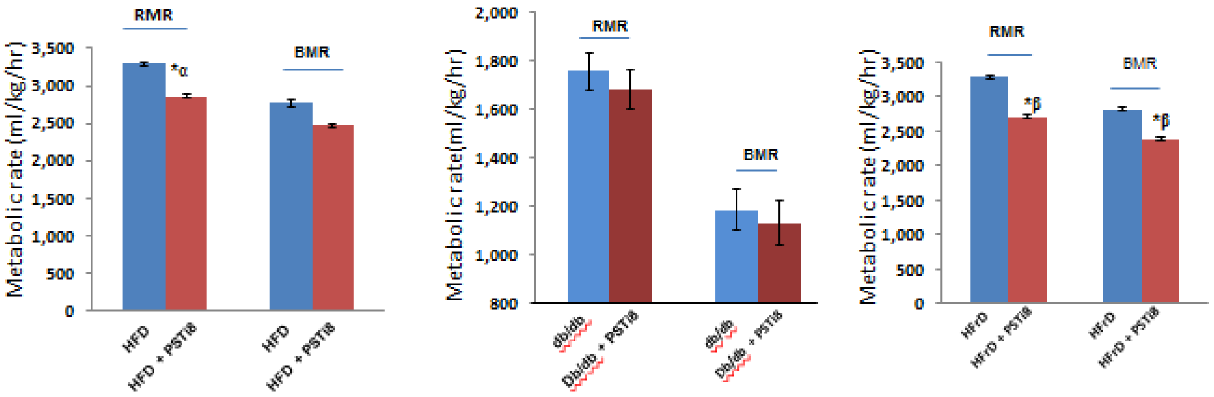

Supplementary Figure 2 Measurement of Basal metabolic rate (BMR) and resting metabolic rate (RMR) in HFD, HFrD-fed and db/db and its PSTi8 treated mice. α, HFD vs HFD +PSTi8; β, HFrD vs HFrD + PSTi8. *, $p<0.05$, error bar ± s.e.m of mean**.**

Supplementary information for figure 5 a, d, g and j (unedited full blots)

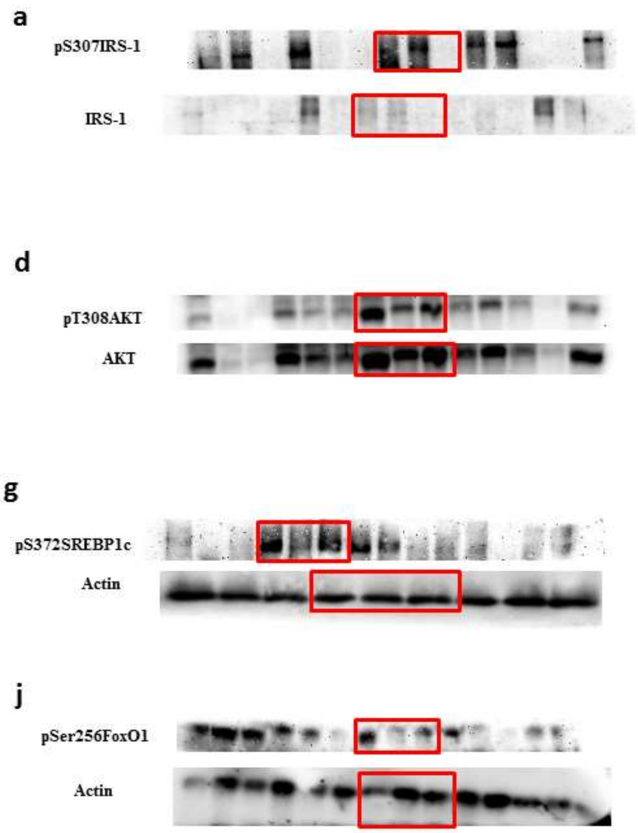

Supplementary information for figure 5 b, e, k and o (unedited full blots)

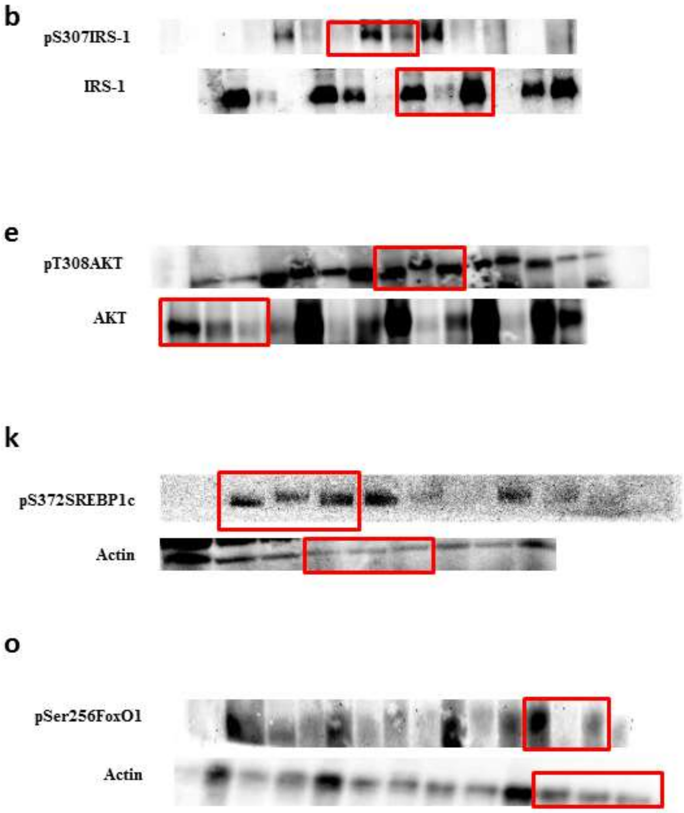

Supplementary information for figure 5 c, f, i and l (unedited full blots)

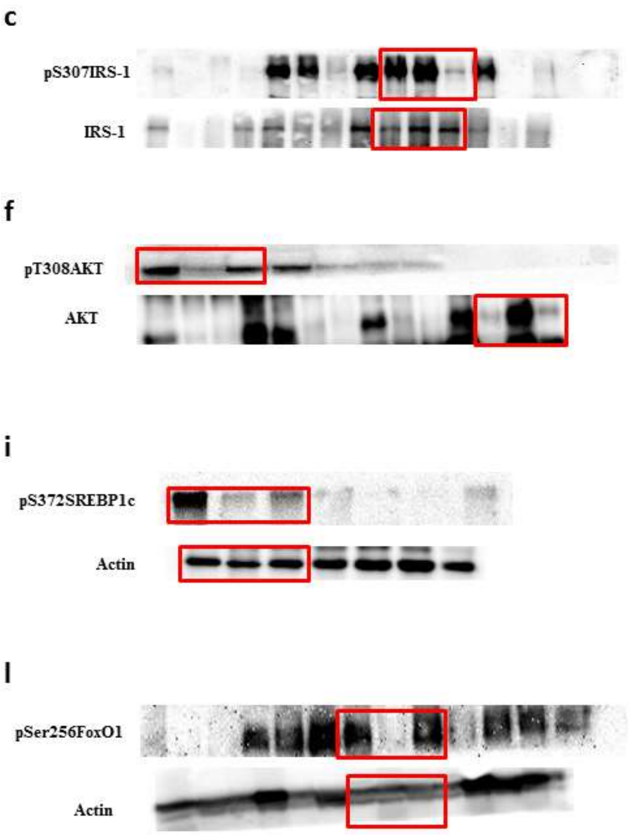

Supplement: Supplementary file 1 — Supplementary File [file 41598_2018_27018_MOESM1_ESM.pdf]
